# Exosomes and Small Extracellular Vesicles as an Alternative to Mesenchymal Stromal Cell Therapy in Knee Osteoarthritis: From Biological Rationale to Clinical Evidence

**DOI:** 10.3390/ijms27093737

**Published:** 2026-04-23

**Authors:** Mahdi Al-Jeabory, Jaroslaw Pecold, Maciej Maslyk, Michal Pruc, Karolina Gromek, Robert Weglowski, Lukasz Szarpak

**Affiliations:** 1 Department of Trauma and Orthopedic Surgery, Silesian Center for Rheumatology, 43-450 Ustron, Poland; 2 Clinical Department of Orthopedics, Faculty of Medical Sciences in Zabrze, Medical University of Silesia, 41-800 Katowice, Poland; 3 Department of Trauma and Orthopedic Surgery, Ruda Slaska City Hospital, 41-793 Ruda Slaska, Poland; 4 Institute of Biological Science, The John Paul II Catholic University of Lublin, 20-950 Lublin, Poland; 5 Institute of Medical Sciences, The John Paul II Catholic University of Lublin, 20-950 Lublin, Polandrobert.weglowski@spzozrp.pl (R.W.); 6 Clinical Department of Trauma and Orthopedic Surgery, The John Paul II Catholic University of Lublin, 21-300 Radzyn Podlaski, Poland; 7 Henry J.N. Taub Department of Emergency Medicine, Baylor College of Medicine, Houston, TX 77030, USA

**Keywords:** biological nanocarriers, extracellular vesicles, GMP manufacturing, Knee osteoarthritis, potency assays, quality control, scalable production, small extracellular vesicles

## Abstract

Knee osteoarthritis (KOA) is a leading cause of pain and disability worldwide, and current treatments remain largely symptomatic, with no disease-modifying therapy established for routine use. This narrative review evaluates extracellular vesicles (EVs) as biological nanocarriers and a cell-free alternative to mesenchymal stromal cell therapy for KOA by examining the biological rationale, preclinical evidence, clinical studies, and current methodological and regulatory requirements. Preclinical findings indicate that EVs may exert immunomodulatory, anti-inflammatory, and chondroprotective effects, supporting their potential to influence joint homeostasis. The review also summarizes current recommendations for EV nomenclature, characterization, and quality control in accordance with the Minimal Information for Studies of EVs 2023 guidelines and highlights key translational challenges, including scalable manufacturing, potency assessment, and regulatory compliance. Clinical evidence to date suggests a favorable safety profile, but efficacy data remain limited and inconsistent; a randomized placebo-controlled trial showed no superiority over placebo, whereas small early human studies suggested possible benefit in selected cases. Overall, EVs represent a promising cell-free strategy for KOA, but current evidence is insufficient to support routine clinical use, emphasizing the need for standardized production, validated potency assays, and robust randomized clinical trials.

## 1. Introduction

Knee osteoarthritis (KOA) is a chronic condition that affects the entire joint, including the cartilage, synovium, subchondral bone, menisci, and surrounding soft tissues. People with KOA often experience pain and stiffness, and joint function can decline over time. This may lower the quality of life and increase healthcare expenses. In 2021, approximately 607 million people worldwide had osteoarthritis, with more than half of these cases being KOA. Among adults aged 70 and older, it ranks as the seventh most common cause of years lived with disability [1,2,3,4,5]. The prevalence is expected to reach 1.1 billion by 2050, with the largest increases likely to occur in low- and middle-income countries [4]. International guidelines consistently recommend non-drug approaches such as patient education, structured exercise programs, and weight loss as the main strategies for KOA management.

The 2019 American College of Rheumatology/Arthritis Foundation, 2022 National Institute for Health and Care Excellence (NICE), and other major professional organizations strongly endorse exercise, weight management in overweight or obese patients, and self-management programs [1,6]. Pharmacological treatments, including topical and oral nonsteroidal anti-inflammatory drugs (NSAIDs) and intra-articular corticosteroid injections, offer symptom relief but do not alter the disease course [1,6,7]. Currently, no disease-modifying osteoarthritis drugs (DMOADs) have been approved for general use. Although preclinical studies suggest that compounds targeting inflammatory cytokines, matrix-degrading enzymes, Wnt signaling, cathepsin K, and nerve growth factor pathways may be effective, clinical trials have not shown consistent improvements in structure or symptoms that meet regulatory approval standards [4,8,9,10]. Because of this, biological therapies remain a promising but debated area of research. Mesenchymal stromal/stem cells (MSCs) have attracted the most interest. Recent systematic reviews and meta-analyses of randomized controlled trials indicate that intra-articular MSC injections may provide modest improvements in pain and function over 3 to 12 months. However, the evidence is limited, and results differ widely based on cell source, preparation, dose, and outcome measures [11,12,13]. Increasing evidence indicates that MSCs primarily exert their effects through paracrine signaling rather than through direct implantation or differentiation. MSCs have immunomodulatory, anti-inflammatory, anti-apoptotic, and chondroprotective effects primarily via the secretion of cytokines, growth factors, and other bioactive molecules [14,15,16].

Extracellular vesicles (EVs) (especially small EVs (sEVs)) are recognized as critical mediators of these paracrine effects, leading to growing interest in EV-based, cell-free therapies. MSC-derived EVs can perform many of the same roles as their parent cells, such as influencing macrophage polarization, reducing inflammation, supporting extracellular matrix production, and affecting subchondral bone remodeling. Compared to cell therapies, they may offer benefits such as lower immunogenicity, greater stability, easier large-scale production, and a lower risk of tumor formation [17,18,19,20]. This review examines the current evidence, the mechanisms of action of EVs, the challenges in their production, and the steps needed to advance MSC-derived sEVs as a novel treatment for KOA.

## 2. Nomenclature and Definitions: Exosomes and Small Extracellular Vesicles

The term exosomes appears frequently in both experimental and clinical research and is often used in place of small extracellular vesicles (sEVs). Still, the International Society for Extracellular Vesicles (ISEV) recommends assigning a specific biogenetic origin only when supported by thorough experimental evidence, which is rarely practical in most studies [21,22].

In the past, EVs were grouped by size and assumed to originate from specific cell types. Exosomes are usually described as vesicles smaller than 150 nm that are released from multivesicular bodies (MVBs) via the endosomal pathway. Larger vesicles, between 100 and 1000 nm, called microvesicles, ectosomes, or shed microvesicles (sMVs), were believed to bud directly from the plasma membrane [22,23]. But this simple division is now considered too basic, since the size ranges often overlap and current isolation methods cannot reliably distinguish vesicles solely by their origin [21,22].

The MISEV2023 guidelines suggest using terms based on physical features and methods of isolation, rather than on their assumed origin [21]. For example, they recommend calling them sEVs (usually under 200 nm) or medium/large EVs (mEVs/lEVs, generally over 200 nm), along with details of the separation method used (sEVs isolated by differential ultracentrifugation). The term exosome should only be used in studies that show strong evidence of endosomal origin, like finding intraluminal vesicles in MVBs with electron microscopy, showing enrichment of endosomal markers (such as CD63, CD81, CD9, TSG101, ALIX), and showing a lack of plasma membrane or other cell compartment markers [21,22].

According to the MISEV2023 recommendations, reliable characterization of EVs should be based on at least three complementary approaches. First, particle concentration and size distribution should be assessed using quantitative techniques such as nanoparticle tracking analysis, tunable resistive pulse sensing, or dynamic light scattering. Second, the vesicular nature of the preparation must be confirmed by demonstrating EV-associated proteins, including at least one membrane- or lipid-bound marker (e.g., CD63, CD81, or CD9), together with a cytosolic protein commonly enriched in EVs, such as TSG101, ALIX, or syntenin. Finally, sample purity should be evaluated by assessing the presence or absence of non-vesicular components, including lipoproteins (apolipoprotein A1 or B) and intracellular contaminants originating from other cellular compartments, such as calnexin from the endoplasmic reticulum, GM130 from the Golgi apparatus, or nuclear proteins like histones [21,24].

In this review, we use the term exosomes to align with common clinical language and historical terminology in cardiac arrest and neurocritical care research. Still, we also note that sEVs are the more accurate and scientific term [21]. When possible, we mention the isolation method and characterization used in the studies we cite to help with understanding and reproducibility. Using both terms makes the review easier for clinicians to understand and still meets ISEV standards, since most clinical studies to date have not conducted the detailed validation needed to confirm a genetic origin [21,25].

Accordingly, throughout this review, the term exosomes is used in a pragmatic clinical sense, while sEVs is used when referring to methodological rigor and compliance with MISEV2023.

## 3. Pathophysiological Rationale for EV-Based Therapy in Knee Osteoarthritis

**Figure 1:**
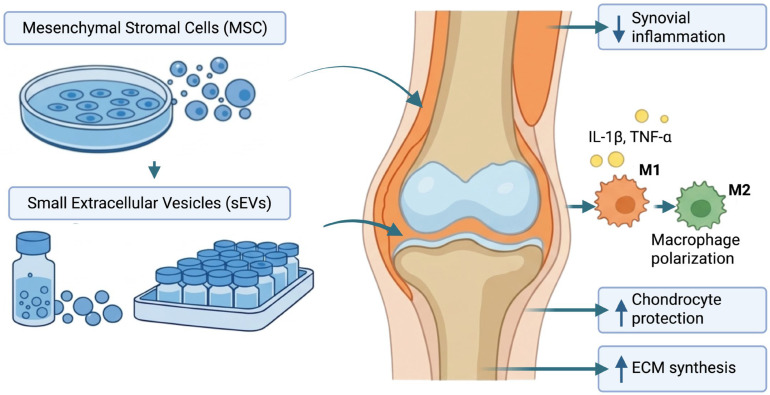
Pathophysiological rationale for extracellular vesicle-based therapy in knee osteoarthritis. Knee osteoarthritis affects the entire joint, including cartilage, synovium, and subchondral bone. Mesenchymal stromal cells (MSCs) exert predominantly paracrine effects through the release of small extracellular vesicles (sEVs), which contain bioactive cargo and act on target cells within the joint microenvironment. MSC-derived sEVs may promote macrophage polarization toward the anti-inflammatory M2 phenotype, reduce synovial inflammation, protect chondrocytes, and support extracellular matrix (ECM) preservation. Created in BioRender. Szarpak, L. (2026) https://BioRender.com/56c8apl.

More and more people are realizing that the mechanical breakdown of cartilage does not cause just KOA but also long-term, low-grade inflammation and problems with communication between tissues that include cartilage, synovium, subchondral bone, and infrapatellar fat pad. Synovial macrophages are essential to this process because they can change into either pro-inflammatory M1 or anti-inflammatory M2 types. M1 macrophages cause synovitis, oxidative stress, and cartilage breakdown by releasing cytokines (IL-1β, TNF-α) and matrix-degrading enzymes (MMPs, ADAMTS-5). M2 macrophages, on the other hand, help tissues heal by releasing TGF-β and IL-10 (Figure 1) [1,26,27,28,29].

KOA arises from complex interactions between joint tissues. Signaling pathways such as TGF-β/Smad, Wnt/β-catenin, RANK/RANKL/OPG, and MAPK facilitate communication between cartilage and bone. Abnormal remodeling of subchondral bone results in increased porosity and angiogenesis, which triggers further inflammatory responses [30,31]. Fundamental are the paracrine interactions between macrophages and chondrocytes, where activated macrophages influence synovial fibroblasts and chondrocytes to produce more matrix metalloproteinases, thus promoting ongoing cartilage degradation [27,28,29,32].

In preclinical models, MSC-derived sEVs have demonstrated their ability to modulate several pathogenic mechanisms in KOA. These include: (1) immunomodulation via macrophage polarization, shifting M1 macrophages to the M2 phenotype by inhibiting NF-κB and MAPK pathways and reducing pro-inflammatory cytokines (IL-1β, TNF-α, IL-6) while increasing anti-inflammatory mediators (IL-10, Arg-1, TGF-β); (2) chondroprotection by preventing apoptosis and senescence, with MSC-sEVs maintaining chondrocyte phenotype by boosting collagen type II and aggrecan production and decreasing IL-1β-induced senescence and apoptosis; (3) preserving extracellular matrix homeostasis by upregulating chondrogenic markers (Sox9, aggrecan, type II collagen) while lowering matrix-degrading enzymes (MMP-13, ADAMTS5); and (4) promoting chondrocyte proliferation and migration, which are vital for cartilage repair [17,33,34,35,36].

The therapeutic cargo of MSC-sEVs includes bioactive molecules such as microRNAs (miR-100-5p, miR-132, miR-153-3p), long non-coding RNAs (lncRNA MEG-3, KLF3-AS1), proteins (CD73, CD5L, TGF-β1, Hsp70), and anti-inflammatory cytokines [36,37,38,39,40]. For instance, exosomes enriched with miR-100-5p safeguard articular cartilage by obstructing mTOR-mediated autophagy pathways, whereas exosomal CD73 facilitates M2 macrophage polarization via adenosine receptor signaling and AKT/ERK activation [38,39]. MSC-sEVs also activate mitochondrial autophagy via the PINK1/Parkin pathway, thereby safeguarding mitochondrial function and reducing oxidative stress in chondrocytes [41].

These observations provide a biological rationale for exploring EV-based therapies as potential disease-modifying interventions for KOA. sEVs have benefits over cell-based MSC therapy, including lower immunogenicity, greater stability, easier storage and handling, no risk of tumorigenicity, and the ability to make targeted changes and incorporate drugs [17,42]. Moreover, reprogramming macrophages from the M1 to M2 phenotype, instead of merely depleting activated macrophages, appears to be an especially efficacious therapeutic strategy, as it can transform the inflammatory microenvironment into a pro-chondrogenic state favorable for cartilage repair [29,32,43].

## 4. Limitations of MSC-Based Therapy and Rationale for Cell-Free Approaches

Although preclinical studies show promising results, MSC-based therapies still face major issues that limit their real-world effectiveness. Clinical trials have demonstrated that MSCs are generally safe. Meta-analyses have found that there is no higher risk of serious side effects, death, or cancer than in controls. However, results for effectiveness have been mixed, and many MSC trials have not met their main goals [44,45,46,47]. As a result, only a few MSC therapies have been approved by regulatory agencies, despite more than 1050 registered clinical trials testing MSCs for a wide range of uses [46,47].

A key challenge is the biological differences among MSCs. These cells can vary widely depending on the donor age, sex, and health; the tissue they come from (such as bone marrow, fat, or umbilical cord); the number of times they have been grown; and the conditions under which they are cultured [44,45,48]. Even MSCs from healthy donors with similar surface markers can behave differently, exhibiting differences in their development, their ability to affect the immune system, and the substances they release [45,48]. This variation leads to batch-to-batch differences and makes it hard to standardize MSC therapies [44,45].

There are also practical and manufacturing challenges that make it harder to use MSCs in the clinic. Producing MSC therapies involves several steps, including isolating, growing, checking quality, freezing, transporting, and delivering live cells on time [44,49]. The cells can lose their function and survival ability during freezing and thawing. After transplantation, only a small number of MSCs survive in the body [44,49,50]. Problems with blood compatibility, such as an immediate blood-mediated inflammatory reaction (IBMIR) after intravenous injection, can trigger immune responses and cause cells to be rapidly removed from the bloodstream [49]. There are also still questions about the best dose, treatment schedule, delivery method, and which patients are most likely to benefit [44,45,47].

While safety issues are usually manageable, they still need to be considered. Serious side effects are uncommon, but possible risks include blockage of small blood vessels due to the cells size (15–30 μm), the risk of spreading infections, and a theoretical risk of tumors, especially if the cells are grown in the lab for a long time [44,49,51]. Surgical procedures also carry their own risks, and there has been one reported death in a case of traumatic spinal cord injury [52].

Recent research suggests that MSCs primarily help the body by releasing signaling molecules rather than by differentiating into new cells or attaching to tissues [18,46,53,54]. Both animal and human studies support the idea that the benefits come from what MSCs secrete. MSCs release a mix of substances, including cytokines, growth factors, chemokines, and EVs that carry proteins, lipids, microRNAs, and mRNAs. These substances help cells communicate and can affect the immune system, inflammation, the growth of new blood vessels, and tissue repair [18,19,54].

**Figure 2:**
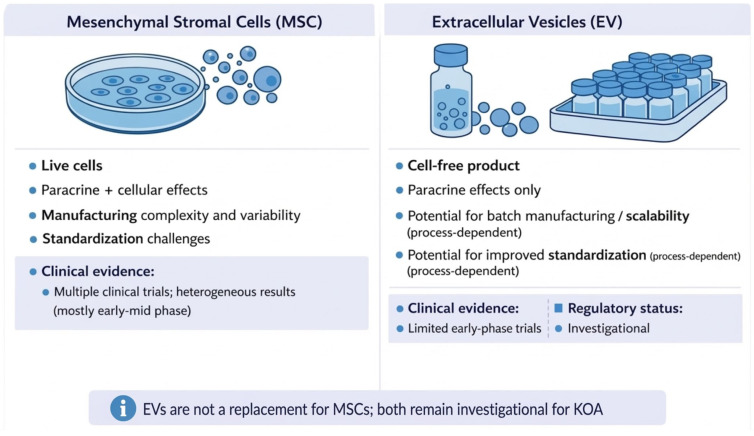
Translational comparison of mesenchymal stromal cell- and extracellular vesicle-based therapies. Mesenchymal stromal cells (MSCs) are live-cell therapies that work through both paracrine and cellular effects. Bringing these therapies to the clinic is challenging due to biological and manufacturing differences, including variations among donors, differences in processing, and issues with standardization. There are also safety concerns related to cell viability and immune responses. Extracellular vesicles (EVs), on the other hand, are cell-free products that only provide paracrine effects. They might be easier to produce in batches and standardize, depending on how they are made. So far, clinical studies on MSCs for knee osteoarthritis have shown mixed results, mostly in early or mid-stage trials. Research on EV therapies is still in the early stages, with only a few first-in-human studies. Currently, both MSC and EV therapies are under investigation for knee osteoarthritis, and no approved products exist. EV therapies are not meant to replace MSC treatments. Created in BioRender. Szarpak, L. (2026) https://BioRender.com/ubd1i2o.

Using MSC-derived EVs instead of whole cells aims to retain MSCs beneficial effects while avoiding some of the main problems with cell-based therapies [19,53,54,55]. MSC-EVs have several possible benefits: (1) they are more consistent, since EVs are a more uniform product than mixed cell groups; (2) they are safer, as they do not carry the same risks of tumor growth, unwanted tissue formation, or blood vessel blockage; (3) they are easier to store, because EVs can be freeze-dried or frozen without worrying about cell survival; (4) they are more compatible with the body and less likely to cause immune reactions, since they do not have MHC molecules and can cross biological barriers more easily; (5) they are easier to use, as they can be kept on hand and do not require the time needed to prepare live cells; and (6) they could be produced on a larger scale, though this still needs better manufacturing methods (Figure 2) [19,50,53,54,56,57].

Studies in animals indicate that MSC-EVs can have effects similar to the original MSCs in models of nerve injury, heart attack, kidney and liver damage, and inflammation. They can replicate the immune-modulating, anti-inflammatory, angiogenic, and tissue-repair functions of MSCs [19,54,55,56]. However, there are still challenges with using MSC-EVs in clinical settings. For instance, it is difficult to determine the correct dose and delivery method, demonstrate their effectiveness in rigorous clinical trials, and develop scalable production methods that meet quality standards [55,56,57].

## 5. Exosomes and sEVs as Cell-Free Therapeutics: Advantages and Limitations

Exosomes and sEVs provide several advantages as cell-free therapies. Their lack of replicative ability removes the risks of uncontrolled growth, tumor formation, and immune rejection associated with cell-based treatments [42,58,59,60]. This safety profile is especially important for intra-articular use in KOA, where maintaining tissue balance is vital [17,59,61]. Manufacturing sEVs is easier than producing living cells because vesicle production, purification, and analysis can be controlled and scaled, ensuring consistency and meeting regulatory standards. sEVs can be customized with specific cargoes (e.g., miRNAs, proteins, drugs) and surface modifications to improve tissue targeting, retention, and therapeutic effectiveness, such as using chondrocyte-specific peptides or charge-reversal strategies for cartilage penetration [17,61,62,63,64,65]. There is still no consensus on the best method for isolating vesicles.

Methods such as ultracentrifugation, size-exclusion chromatography, and precipitation often result in different levels of purity and yield [61,62,63,66,67]. Dosing is also inconsistent, as most studies measure vesicle count, protein levels, or volume, but there is no standard potency test [62,63,67]. Potency and biological activity can vary widely depending on cell source, culture conditions, and vesicle composition [62,67,68]. This variability makes it hard to reproduce results and limits how these findings can be used in clinics. Most evidence for sEVs in KOA comes from small, early trials and preclinical studies. These studies show anti-inflammatory, chondroprotective, and regenerative effects in lab and animal models, but strong data from human trials are still missing [61,62,69].

## 6. Mechanisms of Action of MSC-Derived sEVs

sEVs from mesenchymal stem cells support preclinical models of joint disease in several ways. One significant effect is that they shift macrophages from the pro-inflammatory M1 type to the anti-inflammatory M2 type. This shift lowers the production of IL-1β, IL-6, and TNF-α while increasing IL-10, thereby reducing synovial inflammation and promoting tissue repair. sEVs also inhibit catabolic signaling pathways, such as NF-κB and MAPK [17,33,70,71]. This leads to less production of matrix-degrading enzymes (MMP-13, ADAMTS5) and reduces chondrocyte apoptosis and senescence [17,35].

Increasing the levels of chondrogenic markers (Sox9, aggrecan) helps the extracellular matrix to form. sEVs help form the extracellular matrix by upregulating chondrogenic markers, including Sox9, aggrecan, and type II collagen. They also help chondrocytes proliferate and migrate, thereby repairing cartilage and maintaining the matrix strength. sEVs affect the remodeling of subchondral bone by stopping the formation of osteophytes and other abnormal bone changes. This helps to keep the structure of the joint [17,42,72,73]. These vesicles carry proteins and miRNAs to target cells, which helps drive these effects at the molecular level [14,17,74]. At the molecular level, the biological activity of MSC-derived sEVs appears to depend on the transfer of specific bioactive cargo rather than on vesicle delivery per se. In preclinical osteoarthritis models, selected microRNAs and proteins, including miR-100-5p, CD73, and TGF-β1, have been linked to the regulation of autophagy, the induction of anti-inflammatory macrophage polarization, and the preservation of extracellular matrix homeostasis [38,39,40,41]. Together, these mechanisms reduce inflammation and protect joint structure in preclinical models. This supports the idea that MSC-derived sEVs could help treat osteoarthritis and other joint diseases [17,33,35,42,70,71,73].

## 7. Manufacturing, Characterization, and Quality Control

The MISEV2023 guidelines outline essential steps for translating extracellular vesicle (EV)-based therapies into clinical use. These steps include strict donor screening, robust cell banking, controlled culture conditions, reliable EV isolation and purification, proper formulation and storage, and comprehensive release testing to ensure quality and consistency [21,75,76,77,78,79,80,81].

Donor screening helps reduce variability and the risk of disease transmission. Umbilical cord donors are checked for infectious agents and medical history [75,82,83,84]. Cell banking involves establishing master and working cell banks under cGMP and testing them for identity, sterility, and stability [75,77,82]. Culture conditions, such as the use of xeno-free or serum-free media and control of oxygen levels, directly impact EV yield, content, and potency. Standardizing these conditions is important for consistent batches [75,77,82,83].

Scalable and reliable methods such as tangential flow filtration (TFF) and size-exclusion chromatography (SEC) are recommended for EV isolation and purification, as they offer higher yields and purities than ultracentrifugation [77,79]. Appropriate buffer systems, such as HEPES, and stabilizing agents, including trehalose or BSA, may help preserve EV stability during formulation and storage [80,81]. Storing EVs at −80 °C is best for long-term storage, as repeated freeze–thaw cycles and poor buffers can reduce EV recovery and activity [80,81].

According to MISEV2023, release testing should include several types of checks: measuring particle size and concentration (using nanoparticle tracking analysis or electron microscopy), checking for specific protein markers (like CD9, CD63, CD81, TSG101, ALIX, and making sure calnexin or other non-EV markers are absent), and running functional tests related to the therapy action, such as immunosuppression, wound healing, or anti-inflammatory effects [21,77,85,86,87,88]. Potency tests should reflect the intended biological effect, but assessing them can be difficult because the mechanisms are complex [76,86].

Because the biological activity of EVs is cargo-dependent, variations in donor characteristics, cell source, culture conditions, and isolation workflows may alter the protein, lipid, and RNA composition of EV preparations and thereby influence their therapeutic potency. This heterogeneity represents a major translational challenge, as it may compromise reproducibility, batch-to-batch consistency, and clinical outcomes. Therefore, standardized manufacturing protocols, orthogonal characterization, and mechanism-relevant potency assays are essential for the development of clinically reliable EV-based therapies [75,77,82,85,86].

Early translational studies using cGMP-grade umbilical cord MSC-derived sEVs demonstrate the feasibility of large-scale, standardized production with consistent physicochemical and proteomic profiles, as well as safety and preclinical efficacy [77,82,84]. However, lessons learned include persistent batch-to-batch variability, the need for harmonized potency assays, and the need for further standardization of manufacturing and quality control to meet regulatory requirements [75,77,82,85,86].

## 8. Clinical Evidence for EV-Based Therapies in Knee Osteoarthritis

**Figure 3:**
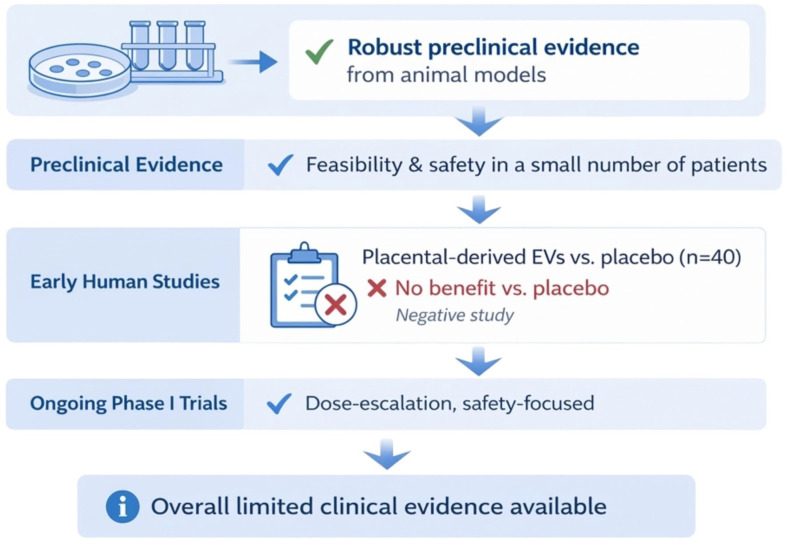
Current clinical evidence for extracellular vesicle-based therapies in knee osteoarthritis. The available evidence for EV-based therapies in knee osteoarthritis includes robust preclinical data, limited early-phase human studies, and a randomized controlled trial of placental-derived extracellular vesicles demonstrating no superiority over placebo. Ongoing phase I clinical trials are primarily focused on safety, feasibility, and dose escalation. Overall, clinical evidence remains limited, and disease-modifying efficacy has not yet been established. Created in BioRender. Szarpak, L. (2026) https://BioRender.com/w6amnde.

Current clinical evidence indicates that extracellular vesicle-based therapies for KOA are safe, although efficacy data remain preliminary and inconsistent (Figure 3). In a randomized, triple-blind, placebo-controlled trial, a single intra-articular injection of placental mesenchymal stromal cell-derived EVs (5 cc, 7 × 10^9^ particles/cc) in patients with grade 2–3 KOA showed no significant improvement in pain, function, or MRI-assessed cartilage structure compared to placebo at 2 and 6 months. However, the study confirmed an excellent safety profile, with no systemic or severe local adverse events [89].

Early-phase clinical studies of cGMP-grade umbilical cord mesenchymal stem cell-derived sEVs (UC-MSC-sEVs) have shown that standardized manufacturing is feasible and intra-articular administration is safe, with no adverse effects reported at 12 months. Preclinical and first-in-human data indicate potential anti-inflammatory and regenerative effects, such as macrophage polarization toward an M2 phenotype, reduced synovitis, and preservation of hyaline cartilage. However, robust clinical efficacy data are still lacking [90,91].

Preclinical and translational studies consistently show that MSC-derived sEVs reduce inflammatory cytokines, promote chondrocyte proliferation and migration, inhibit matrix-degrading enzymes, and support cartilage regeneration [17,33,42,58,60,67,68,91,92,93]. However, in human trials, improvements in pain and function have not consistently exceeded those seen with placebo, and no disease-modifying effects have been confirmed. Overall, extracellular vesicle-based therapies for KOA are safe and biologically active, but their clinical efficacy remains unproven in randomized controlled trials [89,90,91].

## 9. Clinical Relevance for Knee Surgeons

**Figure 4:**
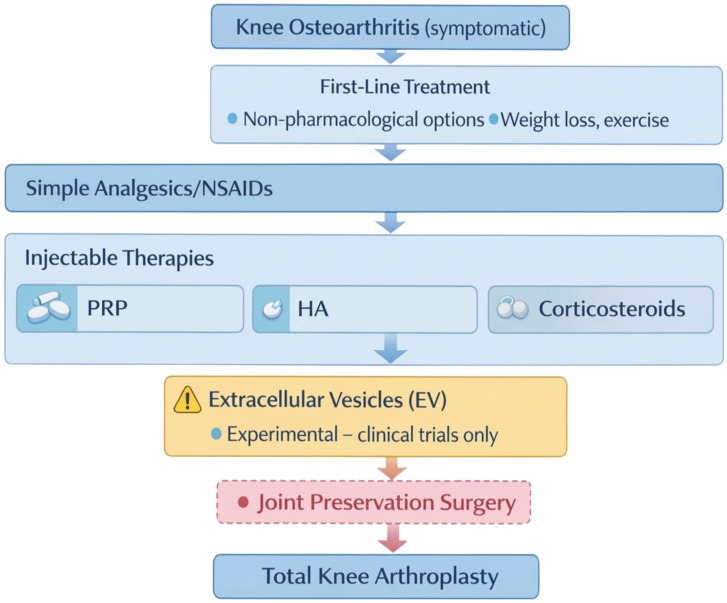
Potential role of extracellular vesicles in clinical decision-making for knee osteoarthritis. Proposed clinical decision-making framework for symptomatic knee osteoarthritis, illustrating established non-pharmacological treatments, pharmacological options, injectable therapies, and surgical interventions. Extracellular vesicles are positioned exclusively as experimental therapies to be considered only within ethically approved clinical trials, reflecting their current investigational status and lack of regulatory approval. Created in BioRender. Szarpak, L. (2026) https://BioRender.com/1cmwz5x.

Current clinical consensus advises against the routine use of exosomes and sEV therapies for KOA. No EV product has shown consistent benefit over placebo in adequately powered randomized clinical trials, and no exosome or EV therapy has received regulatory approval for intra-articular use in osteoarthritis in the US or Europe (Figure 4) [17,59,62].

Compared to established intra-articular therapies such as glucocorticosteroids, hyaluronic acid, platelet-rich plasma (PRP), and MSC-based cell therapies, EVs have a favorable safety profile and immunomodulatory potential. However, they have not demonstrated clinical efficacy in randomized trials for reducing pain, improving function, or regenerating cartilage [17,59,60,62]. Glucocorticosteroids and hyaluronic acid provide short-term pain relief; PRP shows moderate efficacy in selected populations; and MSCs are considered experimental, with regenerative potential but limited availability and variable results [17,60,62].

The main barriers to implementing EVs in orthopedic practice include a lack of standardization in isolation and dosing methods, variability in composition and biological activity, the absence of recognized potency tests, limited clinical data, and an unregulated legal status [42,59,62,67]. Additionally, EVs do not outperform current therapies in clinical efficacy, and their use remains experimental, limited to early-phase studies [17,59,62].

Exosomes and sEVs are promising but are not currently recommended as alternatives to established intra-articular therapies for KOA. Their routine use requires further clinical research, standardization, and regulatory approval [17,59,67].

## 10. Conclusions

Exosomes and other sEVs are promising cell-free treatment options for KOA and could serve as realistic alternatives to MSC-based therapies. Still, from an orthopedic and surgical perspective, EV-based therapies are not yet ready for routine clinical use. Before EVs can be used responsibly to treat KOA, we need well-designed randomized trials, consistent manufacturing processes, reliable potency testing, and regulatory approval. At present, EVs should be viewed as a promising translational concept rather than a clinically actionable therapy for KAO. From a nanobiotechnology perspective, EVs represent biological nanocarriers with therapeutic potential, but their clinical implementation is currently limited by unresolved challenges in large-scale manufacturing, batch-to-batch reproducibility, potency testing, and regulatory standardization.

## Data Availability

No datasets were generated or analyzed during the current study. All data discussed in this review are available in the published literature cited in the manuscript.

## Abbreviations

The following abbreviations are used in this manuscript:

ALIX	ALG-2-interacting protein X
Arg-1	Arginase 1
BSA	Bovine serum albumin
CD5L	CD5 molecule-like protein
CD9	Cluster of differentiation 9
CD63	Cluster of differentiation 63
CD73	Cluster of differentiation 73
CD81	Cluster of differentiation 81
cGMP	Current Good Manufacturing Practice
DMOADs	Disease-modifying osteoarthritis drugs
ECM	Extracellular matrix
ERK	Extracellular signal-regulated kinase
EVs	Extracellular vesicles
GM130	Golgi matrix protein 130
HEPES	4-(2-hydroxyethyl)-1-piperazineethanesulfonic acid
Hsp70	Heat shock protein 70
IBMIR	Instant blood-mediated inflammatory reaction
IL-1β	Interleukin 1 beta
IL-6	Interleukin 6
IL-10	Interleukin 10
ISEV	International Society for Extracellular Vesicles
KOA	Knee osteoarthritis
lEVs	Large extracellular vesicles
lncRNA	Long non-coding RNA
MAPK	Mitogen-activated protein kinase
mEVs	Medium extracellular vesicles
miRNA	MicroRNA
MISEV2023	Minimal Information for Studies of Extracellular Vesicles 2023
MMP-13	Matrix metalloproteinase 13
MSCs	Mesenchymal stromal/stem cells
mTOR	Mechanistic target of rapamycin
NF-κB	Nuclear factor kappa-light-chain-enhancer of activated B cells
NICE	National Institute for Health and Care Excellence
OA	Osteoarthritis
OPG	Osteoprotegerin
PINK1	PTEN-induced kinase 1
PRP	Platelet-rich plasma
RANK	Receptor activator of nuclear factor kappa-B
RANKL	Receptor activator of nuclear factor kappa-B ligand
SEC	Size-exclusion chromatography
sEVs	Small extracellular vesicles
sMVs	Shed microvesicles
Sox9	SRY-box transcription factor 9
TFF	Tangential flow filtration
TGF-β	Transforming growth factor beta
TNF-α	Tumor necrosis factor alpha
TSG101	Tumor susceptibility gene 101
